# Functional neurologic disorders in an adult with propionic acidemia: a case report

**DOI:** 10.1186/s12888-021-03596-2

**Published:** 2021-11-22

**Authors:** Alexis Tarrada, Solène Frismand-Kryloff, Coraline Hingray

**Affiliations:** 1 Service de Neurologie, CHRU Central Nancy, 54000 Nancy, France; 2 Faculté de Médecine, Université Paris Descartes, 75006 Paris, France; 3 Centre Psychothérapique de Nancy, Pôle Universitaire du Grand Nancy, 54000 Laxou, France

**Keywords:** Propionic acidemia, Psychiatry, Metabolic disorder, Psychiatric profile, Case report

## Abstract

**Background:**

Inborn errors of metabolism are often characterized by various psychiatric syndromes. Previous studies tend to classify psychiatric manifestations into clinical entities. Among inborn errors of metabolism, propionic acidemia (PA) is a rare inherited organic aciduria that leads to neurologic disabilities. Several studies in children with PA demonstrated that psychiatric disorders are associated to neurological symptoms. To our knowledge, no psychopathological description in adult with propionic acidemia is available.

**Case presentation:**

We aimed to compare the case of a 53-year-old woman with PA, to the previous psychiatric descriptions in children with PA and in adults with other inborn errors of metabolism. Our patient presented a large variety of signs: functional neurologic disorders, borderline personality traits (emotional dyregulation, dissociative and alexithymic trends, obsessive-compulsive disorders), occurring in a context of neurodevelopmental disorder.

**Conclusion:**

Clinical and paraclinical examinations are in favor of a mild mental retardation since childhood and disorders of behavior and personality without any definite psychiatric syndrome, as already described in other metabolic diseases (group 3). Nonetheless, further studies are needed to clarify the psychiatric alterations within adult patients with PA.

## Background

Propionic Acidemia (PA) is a rare inherited (recessive autosomal) organic aciduria due to a propionyl-CoA carboxylase activity deficiency [4]. The disease is characterized by acute metabolic episodes, cardiac and renal failures and neurologic disorders [8]. Chronic neurologic and cognitive complications are frequent: movement disorders, spastic paresis, intellectual disability and strokes of basal ganglia [8]. There are two forms of the disorder: a severe form occurring in early life and associated with a high mortality rate and a chronic form associated with an evolution into neurologic disabilities [4].

Neurocognitive complications of organic acidurias are well described [13, 18–20]. However, literature about psychiatric symptoms occurring in organic acidurias is relatively scarce, less detailed and focuses mostly on children. Nonetheless, results showed that patients would be more susceptible to develop behavioral disorders such as self-damaging or self-regulating issues, anxious and avoidant behaviors, confirmed by parents [11]. Those behavioral signs are concordant with the higher prevalence of neurodevelopmental disorders in organic acidurias [18]. Concerning PA, the most frequent disorders are neurodevelopmental disorders from the Autism Spectrum ([5, 21]. A case series of 19 patients, aged between 2 and 25 years described 2 patients with typical autism, 2 patients with other autism spectrum disorders and 5 others with a broader autism phenotype (de la [3]). They also reported three patients with attention deficit symptoms and two with significant chronic anxiety symptoms. In addition, 13 of those 19 patients presented an intellectual disability. Rarely, psychotic signs are also reported: visual hallucinations [17] and rarely acute psychosis [6]. As compared to other organic acidurias, patients with PA would be more likely to develop psychiatric manifestations, such as attention deficit, or psychotic state [13, 20]. Unfortunately, those studies mostly concentrated on children or young adults. Hence, literature does not provide the psychiatric profile or evolution in adults suffering from Propionic Acidemia, neither other organic acidurias.

However, psychiatric manifestations have been described in adults suffering from other inborn errors of metabolism, and classified into three groups [16]. Group 1 included emergencies revealed by recurrent attacks of delusional confusion and concerned: urea cycle defects, homocysteine remethylation defects and porphyrias. Group 2 consisted in schizophrenia-like syndrome arising in young adults more often associated with catatonia, visual hallucinations and deterioration with treatments, and concerned: homocystinurias, Wilson disease, neurolipidosis (Niemann-Pick C, adrenoleukosdystrophy…). Group 3 included patients with mild mental retardation since childhood and disorders of behavior or personality with no definite psychiatric syndrome, and concerned: homocystinurias, cerebrotendinous xanthomatosis, nonketotic hyperglycinaemia, monoamine oxidase A deficiency, succinic semialdehyde dehydrogenase defects, creatine transporter deficiency, and α- and β-mannosidosis. But no case of propionic acidemia was described among those groups, that included metabolic disorders with a pathophysiology distinct from organic acidurias.

Thus, we based our clinical evaluation of a 53-year-old woman with PA, on pedopsychiatric descriptions, available for PA and organic acidurias, to get an idea of the evolution of childhood disorders. We also aimed to compare the psychiatric evaluation, to the psychiatric manifestations described in other inborn errors of metabolism in adults.

## Case presentation

The patient is a 53-year-old woman diagnosed with Propionic Acidemia (result of genetic mutation not available) at the age of 3 in the context of an acute metabolic episode revealing by a right-sided hemiplegia. She had a second acute episode revealed by a left-sided hemiparesis at 9 years old. Then she was well-controlled by a low-protein regimen.

She was followed by a neurologist for functional neurologic disorder appeared around the age of 50 years old, such as dystonia, dysphonia or abrupt loss of muscular tonus in legs. All those disorders improved during the follow-up: she had no dysphonia or dystonia anymore, but still presented rare episodes of muscular weakness. A psychiatric assessment was needed to complete the neurological evaluation.

### Psychiatric examination

She had multiple follow-ups by child-psychiatrist for behavior disorders such as clastic crisis and multiple scarifications. She was hospitalized once around 25 years old for a suicidal attempt (self-phlebotomy). She also reported that psychiatrists suspected a bipolar disorder given cyclothymic signs and was treated by Aripiprazole. After 2 to 3 years, it was replaced by Venlafaxine.

She also reported traumatic events in her life: sexual abuse by a first husband, emotional and physical abuse by a second husband. We noted possible emotional neglect in childhood (communication issues with her parents).

She had been passionate by Asian culture (Korea and Japan) since this age of 5 years old and had correspondents with whom she shared her interest. She has had lots of pets and a reborn baby to bring herself some affection and to cope with a feeling of loneliness.

She stopped her psychiatric follow-up for at least 6 years but kept taking her antidepressant, Venlafaxine 225 mg/day and anxiolytic, Alprazolam when needed (medications were reconducted by her general practitioner).

On examination, she was mildly desinhibited and logorrheic, but her mood was globally stable. There was no argument for a hypomaniac, maniac neither depressed syndrome. She reported lots of bodily preoccupations during childhood and teenage with panic attacks (focused on nudity) that have progressively disappeared. Currently, she has been presenting an anxious apprehension of potential muscular weakness: she systematically went out with her wheelchair, although she has never needed it. She also has suffered from obsessive-compulsive disorders for several years. For medical appointments, she systematically established a very-detailed tables describing her symptoms (neurologic, psychiatric…) day per day for the last 6 months…The time dedicated to this tables had consequences on her sleep, her housekeeping and her personal hygiene.

She presented an emotional dysregulation with impulsivity, affective fluctuations and self-induced mutilations. The reborn baby seemed to satisfy an affective emptiness. She also had an alexithymia and dissociative symptoms with depersonalization and multiple episodes of dissociative amnesia in concordance with her traumatic history. During the current follow-up, she was once more victim of a sexual assault that has triggered a sudden episode of dissociative amnesia. She lost memory of the 4 past years and did not recognize her house, her doctor neither her dogs. She was completely detached when she told about the aggression. This dissociative episode has spontaneously recovered after 1 week.

Autistic symptoms were not clinically marked: we only found difficulties for understand implicit sense and some sensorial intolerance. She had no psychotic sign: no hallucination, no delusion or disorganized thoughts. We did not find symptoms of eating-behavior disorder.

### Psychometric evaluation

Table 1.

**Table 1:** Summary of psychometric scores of the patient

Scale	Subscore	Total score	Interpretation
TAS-20	Difficulty in identifying feelings: 26 Difficulty in describing feelings: 12 Outward-looking thinking: 18	Total of 56	Highly probable alexithymia
DES	Absorption in imaginary: 32.8 Depersonalization/ Derealization: 14.2 Dissociative amnesia: 13.6	Total of 21.4	Moderate dissociative tendency
GAD-7		Total of 16	High probability of anxious disorder
Y-BOCS	Obsession score: 0 Compulsion score: 11	Total of 11	Mild obsessive-compulsive disorder

*TAS* Toronto Alexithymia Scale, *DES* Dissociative Experience Scale, *GAD* Generalized Anxiety Disorder; *Y-BOCS* Yale-Brown Obsession-compulsion scale

#### Neuropsychological evaluation

The patient presented a mild global intellectual disability. On memory functions she had deficiencies in short-term memory and working memory, but episodic memory was intact. Executive functions were globally preserved. She also had difficulty in planification and a longer processing speed (Table 2).

**Table 2:** Resume of neuropsychologic assessment from Wechsler Adult Intelligence Scale

Function	Score	Interpretation
Total Intellectual Quotient	83	moderate to low efficiency
General Intellectual Ability	91	
Verbal Comprehension Index	102	preserved
Perceptual Reasoning Index	82	Middle low
Processing Speed Index	81	Middle low
Working Memory Index	77	Altered

#### Brain magnetic resonance imaging

**Fig. 1:**
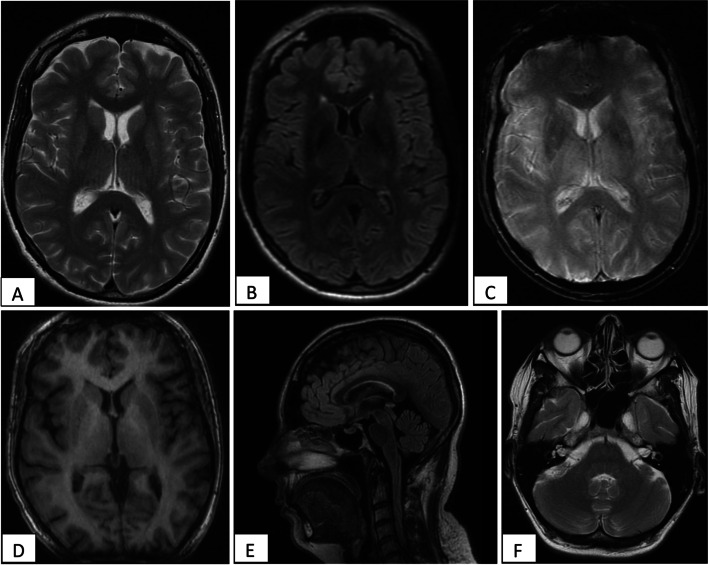
Brain Magnetic Resonance Imaging of the patient, revealing absence of abnormalities especially within basal ganglia. **A** Axial T2 sequencing centered on basal ganglia; **B** Axial FLAIR sequencing centered on basal ganglia; **C** Axial T2* centered on basal ganglia; **D** Axial T1 sequencing centered on basal ganglia; **E** Sagittal FLAIR sequencing centered on midbrain; **F** Axial T2 sequencing centered on cerebellum)

In front of the presence of motor dysfunction and cognitive deficiency, a brain MRI was performed. Furthermore, given the obsessive-compulsive syndrome, we looked for possible attempts of basal ganglia due to propionic acidemia. Indeed in less than 10% of cases, obsessive-compulsive disorders are secondary to basal ganglia pathology [9, 10]. Here, MRI did not highlight significant abnormality (Fig. 1).

## Discussion and conclusion

Our objective was to compare the psychiatric evaluation of an adult with propionic acidemia to other organic acidurias and inborn errors of metabolism and to get an idea of the psychopathological evolution of psychiatric childhood disorders of propionic acidemia.

In our patient, psychiatric and neuropsychological examinations found a mild mental retardation associated with features evoking a “borderline-like” personality disorder with emotional dysregulation (that had led to a temporary suspicion of bipolar disorder), dissociative and alexithymic symptoms and self-induced injuries. Dissociative and alexithymic symptoms were clinically patent and potentially explained her functional neurological signs, even though psychometric scores rated below the certitude threshold (> 61 for TAS and > 30 for DES) [2, 7]. She also had a mild Obsessive-Compulsive Disorder, confirmed by Y-BOCS. In resume, the patient suffered from several psychiatric symptoms from different syndroms, associated with behavioral disorders and mild intellectual disability. All those results are in favor to a psychiatric profile corresponding to the group 3 described previously [16]. However, given the lack of publication on psychiatric manifestations in adult with propionic acidemia, we do not know whether our case is representative or not. Secondly, the psychiatric manifestations of our patient could be due to her traumatic history and not to her metabolic disorder. Indeed, borderline personality traits and dissociative symptoms are associated with sexual and physical abuse and emotional neglect (de Aquino [1]). In addition, one could suppose that propionic acidemia is associated with an emotional dysregulation, as suggested by previous literature in children and young adults with PA or others organic acidurias [11, 13, 18]. This issue of self-regulation could lead to an hyperreactivity to traumatic events. This hypothesis might explain the high arousal of dissociative amnesia presented by our patient. But further evaluations of adult with propionic acidemia need to be performed to get a more precise representation of the psychiatric profile and to add in the classification developed by Sedel et al. As a result, different metabolic diseases with miscellaneous pathophysiology could lead to the same psychiatric phenotype. One could assume, either that the psychiatric symptoms occurring in metabolic disorders are not specific to it but are rather a comorbidity of a chronic condition; or that different metabolic pathways are involved in the same neurotransmitter regulation [15].

Regarding the psychopathological evolution, anamnestic investigations did not find relevant arguments for a complete autism but the patient presented a corporal anxiety in childhood (that were described in the serie of cases (de la [3]), a difficulty to understand implicit and sensorial intolerances. Moreover, she developed an obsessive-compulsive disorder that is not due to basal ganglia lesions. We can then suppose that it could be an equivalent or an evolution of rituals and stereotypies of an autism spectrum disorder. But since we did not have access to the pedopsychiatric data of our patient, this hypothesis could not be verified. Further clinical investigations of adult with propionic acidemia could help to draw a psychopathological evolution from childhood to adulthood.

Finally, given the risk of basal ganglia strokes in propionic acidemia, a brain MRI should be systematically prescribed in front of Obsessive-compulsive disorders that can be secondary to basal ganglia dysfunction [9, 10, 14].

This clinical observation highlights the importance of a psychiatric assessment in adult patients with metabolic disorders, whereas literature is relatively scarce about this topic. Yet, metabolic disorders are known to be a cause of secondary psychiatric disorders [12, 15], mental disorders are frequent comorbidities of metabolic diseases, and could be useful in research as a comprehensive model for primary psychiatric disorders [15].

## Data Availability

The datasets used and/or analysed during this study concerned confidential data and are available only from the corresponding author on reasonable request.

## Abbreviations

PA: Propionic Acidemia
mg: Milligrams
MRI: Magnetic Resonance Imaging
TAS: Toronto Alexithymia Scale
DES: Dissociative Experience Scale
GAD: Generalized Anxiety Disorder
Y-BOCS: Yale-Brown Obsession-compulsion scale
