# Correction to: Functional neurologic disorders in an adult with propionic acidemia: a case report

**DOI:** 10.1186/s12888-021-03656-7

**Published:** 2022-01-10

**Authors:** Alexis Tarrada, Solène Frismand-Kryloff, Coraline Hingray

**Affiliations:** 1 Service de Neurologie, CHRU Central Nancy, 54000 Nancy, France; 2 Faculté de Médecine, Université Paris Descartes, 75006 Paris, France; 3 Centre Psychothérapique de Nancy, Pôle Universitaire du Grand Nancy, 54000 Laxou, France

Following the publication of the original article [1], the authors identified that the funding note was missing. The funding note is given below.

Funding

PHRC EDUQ CPNE paid for the language editing service, and contributed in writing the manuscript.

The original article [1] has been corrected.
